# Short-Term Effects of Meloxicam and Marine-Based Fatty Acid Compounds on Objective Limb Loading and Owner-Reported Outcomes in Dogs with Hip Osteoarthritis: A Randomized, Double-Blind, Placebo-Controlled Trial

**DOI:** 10.3390/ani16213435

**Published:** 2026-10-30

**Authors:** Narudee Kashemsant, Panida Panitsillapakit, Monchanok Vijarnsorn, Chalermpol Lekcharoensuk, Sarawut Yangtara, Julaporn Pooliam, B. Duncan X. Lascelles

**Affiliations:** 1 Department of Physiology, Faculty of Veterinary Medicine, Kasetsart University, Bangkok 10900, Thailand; narudee.k@ku.th; 2 Department of Companion Animal Clinical Sciences, Faculty of Veterinary Medicine, Kasetsart University, Bangkok 10900, Thailand; panda1982panida@gmail.com (P.P.); fvetcpl@ku.ac.th (C.L.); great_vt14@hotmail.com (S.Y.); 3 Siriraj Research Data Management Unit, Research Department, Faculty of Medicine Siriraj Hospital, Mahidol University, Bangkok 10700, Thailand; julaporn.poo@mahidol.ac.th; 4 Translational Research in Pain and Comparative Pain Research and Education Center, Department of Clinical Sciences, College of Veterinary Medicine, North Carolina State University, Raleigh, NC 27607, USA; dxlascel@ncsu.edu; 5 Center for Translational Pain Research, Department of Anesthesiology, Duke University, Durham, NC 27710, USA; 6 Thurston Arthritis Center, UNC School of Medicine, Chapel Hill, NC 27599, USA

**Keywords:** canine hip osteoarthritis, peak vertical force, force-plate gait analysis, meloxicam, marine-derived fatty acids, green-lipped mussel, responder analysis, owner-reported outcome measures

## Abstract

Canine hip osteoarthritis (OA) requires multidimensional assessment. This randomized, double-blind, placebo-controlled trial evaluated 4-week treatment with meloxicam, PCSO-524, EAB-277, meloxicam plus PCSO-524, or placebo in 203 client-owned dogs with naturally occurring hip OA. The principal outcome was index-hindlimb peak vertical force (PVF) measured by force-plate gait analysis. Secondary outcomes included PVF responder status, orthopedic assessment score (OAS), Liverpool Osteoarthritis in Dogs (LOAD), Canine Brief Pain Inventory (CBPI), and safety assessments. The primary baseline-adjusted mean PVF analysis did not demonstrate treatment superiority among groups. A secondary responder analysis using an analysis-stage >5%BW PVF threshold showed that meloxicam had a nominally higher Week 4 responder probability than placebo (relative risk = 4.000; 95% confidence interval, 1.223 to 13.079; *p* = 0.022), interpreted as supportive rather than confirmatory. Within-group score reductions were observed in LOAD and CBPI pain interference for the combination group, LOAD for PCSO-524, and CBPI pain interference for EAB-277. OAS did not change significantly within any group. Between-group differences in LOAD and CBPI changes were not significant. No consistent group-level laboratory safety concern was detected. These findings support multidimensional assessment while emphasizing that secondary results do not establish treatment superiority.

## Simple Summary

Dogs with hip osteoarthritis often have joint pain and decreased mobility, but it can be difficult to know whether a treatment is truly helpful. Using several assessment tools may provide a more complete picture of improvement after treatment. This study compared meloxicam, two marine-based fatty acid products, a combination of meloxicam with one marine-based product, and placebo treatment in client-owned dogs with naturally occurring hip osteoarthritis over 4 weeks. We assessed weight bearing on the affected hindlimb, veterinarian-assessed orthopedic status, owner-reported mobility and pain-related daily activity, and short-term blood safety results. Average weight-bearing measurements did not clearly differ among treatment groups. However, dogs receiving meloxicam were more likely than placebo-treated dogs to reach a conservative individual-dog weight-bearing improvement threshold at Week 4. Combination treatment showed improvements in owner-reported mobility and pain-related daily activity. One marine-based product showed owner-reported mobility improvement, whereas the other showed owner-reported improvement in pain-related daily activity. These owner-reported findings were not matched by clear objective evidence of better weight bearing. No consistent group-level safety concern was detected in blood test results, although individual clinically relevant events occurred. These findings may help veterinarians and owners judge treatment response using complementary tools rather than a single measure.

## 1. Introduction

Osteoarthritis (OA) is a common chronic musculoskeletal disorder in dogs and is characterized by progressive deterioration of joint tissues, pain, impaired mobility, and reduced quality of life [1]. Prevalence estimates vary according to age, breed, case definition, and study population, but OA is recognized as an important cause of chronic pain and functional impairment in companion dogs [2,3]. Hip OA is clinically important because pain and impaired hindlimb function can reduce weight-bearing, mobility, exercise tolerance, and daily activity. Because OA is managed rather than cured, treatment goals focus on reducing pain, improving limb function, preserving mobility, and maintaining quality of life [1,4].

Assessment of treatment response in canine OA is challenging because OA affects multiple dimensions of pain and function. Veterinarian-based assessments, including orthopedic assessment scores (OAS), provide clinical evaluation of lameness, joint mobility, and pain on manipulation [5,6]. Owner-reported clinical metrology instruments, such as the Liverpool Osteoarthritis in Dogs (LOAD) questionnaire and the Canine Brief Pain Inventory (CBPI), capture owner-perceived mobility, pain severity, pain-related interference with daily activities, and quality-of-life impact [7,8,9,10]. These instruments are clinically valuable because owners observe their dogs during routine activities in the home environment. However, owner-reported outcomes may be influenced by caregiver expectations, contextual effects, and interpretation of questionnaire items [11]. Objective gait analysis can therefore provide complementary information. Ground reaction forces, particularly peak vertical force (PVF), are widely used objective measures of limb-loading function in dogs with OA and are less directly affected by caregiver perception than owner-completed questionnaires [12,13].

Non-steroidal anti-inflammatory drugs (NSAIDs) remain a cornerstone of medical management for canine OA pain. Meloxicam, an oxicam NSAID with preferential cyclooxygenase-2 inhibitory activity, is widely used for the management of pain and inflammation associated with acute and chronic musculoskeletal disease in dogs [14]. Previous clinical trials have reported improvements in lameness and other clinical signs in dogs with OA treated with meloxicam [15,16]. However, OA often requires long-term management, and NSAID therapy may be associated with gastrointestinal, renal, hepatic, or other adverse effects, particularly in susceptible dogs or during prolonged use [17]. These concerns support a multimodal approach to OA management that integrates pharmacologic analgesia, weight control, activity modification, rehabilitation, nutritional support, and appropriate monitoring [1].

Marine-based fatty acid compounds have received increasing attention as potential adjunctive or alternative interventions for canine OA. These products include lipid extracts derived from New Zealand green-lipped mussel (*Perna canaliculus*) and formulations that combine green-lipped mussel lipid fractions with krill oil [18,19,20,21]. They contain multiple lipid components, including long-chain polyunsaturated omega-3 fatty acids, and are proposed to modulate inflammatory pathways through omega-3 fatty acid-related mechanisms. This provides a biologically plausible rationale for evaluating their clinical effects in dogs with OA, particularly as part of multimodal management.

Clinical evidence for marine-based fatty acid compounds in canine OA is developing but remains incomplete. NSAIDs remain a cornerstone of medical management for canine OA pain, and omega-3-containing nutraceuticals are commonly considered adjunctive options in multimodal OA management [22,23]. Previous studies have evaluated NSAIDs, green-lipped mussel-derived products, fish oil-based interventions, and other nutraceuticals using a range of clinical, owner-reported, and objective gait out-comes [20,24,25]. However, available studies differ in treatment comparator, treatment duration, outcome measures, and study design, making direct clinical interpretation difficult.

A specific evidence gap remains regarding the short-term comparative performance of meloxicam, marine-based fatty acid compounds, their combination, and placebo when evaluated within the same randomized, double-blind protocol. In particular, limited evidence is available on whether adding PCSO-524 to meloxicam provides additional objective or owner-perceived benefit compared with meloxicam or PCSO-524 alone. There is also limited integrated evidence comparing group-level mean PVF, in-dividual-dog PVF responder status, veterinarian-assessed orthopedic findings, owner-reported LOAD and CBPI outcomes, and short-term laboratory safety findings within the same trial. Because outcome measures in canine OA studies capture different dimensions of pain, function, and treatment response, this multidimensional approach may provide a more clinically useful interpretation than reliance on a single outcome measure [26]. The present five-group design was therefore intended to address this evidence gap by comparing meloxicam, PCSO-524, EAB-277, meloxicam plus PCSO-524, and placebo in client-owned dogs with naturally occurring hip OA.

Therefore, the primary objective of this study was to evaluate the short-term efficacy of meloxicam, two marine-based fatty acid compounds, a combination treatment with meloxicam and a marine-based fatty acid compound against placebo in client-owned dogs with naturally occurring hip OA, using PVF of the index hindlimb as the primary objective outcome. Secondary objectives were to evaluate PVF responder status, veterinarian-assessed OAS, owner-reported LOAD and CBPI outcomes, and short-term hematological and serum biochemical safety variables. We hypothesized that active interventions would improve PVF compared with placebo. We further hypothesized that combination treatment with meloxicam and a marine-based fatty acid compound would provide additional clinical benefit compared with either treatment alone.

## 2. Materials and Methods

### 2.1. Ethical Approval and Study Setting

The study protocol was reviewed and approved by the Institutional Animal Care and Use Committee of the Faculty of Veterinary Medicine, Kasetsart University (approval no. ACKU61-VET-018). Client-owned dogs presented to the Kasetsart University Veterinary Teaching Hospital, Bang Khen Campus, Bangkok, Thailand were enrolled between January 2019 and August 2021.

Before enrollment, owners received written information describing the study objectives, procedures, potential benefits, and foreseeable risks. Written informed consent was obtained from each owner. Participation was voluntary, and owners were permitted to withdraw their dogs from the study at any time.

### 2.2. Study Design and Participants

This was a randomized, double-blind, placebo-controlled, parallel-group clinical trial evaluating five treatment regimens administered for 4 weeks to client-owned dogs with naturally occurring hip osteoarthritis.

Client-owned medium- to large-sized dogs of either sex were recruited from the Kasetsart University Veterinary Teaching Hospital. Eligible dogs were required to have clinical and radiographic evidence of hip osteoarthritis, as detailed below.

### 2.3. Eligibility Criteria

Dogs were eligible for enrollment if they were medium- to large-sized dogs of either sex, at least 18 months of age, weighed 18 to 50 kg, and had clinical and radiographic evidence of hip osteoarthritis. Clinical signs consistent with hip osteoarthritis included one or more of the following: hindlimb lameness while walking or running, difficulty rising or sitting, hindlimb muscle atrophy, or reduced hip joint range of motion. Eligible dogs were also required to have pain localized to at least one hip joint during orthopedic examination, radiographic evidence of degenerative changes in at least one hip joint, hematologic and serum biochemical results considered clinically acceptable for study participation, and the ability to run successfully across the force-plate runway.

Dogs were excluded if they had forelimb lameness, neurologic abnormalities, clinically relevant concurrent systemic disease, or clinically important concurrent orthopedic abnormalities affecting gait, including cranial cruciate ligament rupture, patellar luxation, or instability of another joint. Dogs were also excluded if they had a previous condition or surgical procedure that could substantially affect walking or running ability, including femoral head and neck excision; orthopedic surgery within the preceding 8 months; surgery for cranial cruciate ligament rupture within the preceding 6 months; or pregnancy or lactation.

Dogs previously receiving nutraceutical products or nonsteroidal anti-inflammatory drugs underwent a washout period of at least 2 weeks before enrollment. Corticosteroids and injectable sodium pentosan polysulfate were discontinued for at least 4 weeks before enrollment.

#### Radiographic Assessment for Eligibility

Radiographs were obtained during screening to confirm radiographic evidence of hip osteoarthritis in dogs with compatible clinical signs and hip pain on orthopedic examination. Standard orthogonal radiographic views, including lateral and ventro-dorsal views of the hip/pelvic region, were obtained. Radiographs were evaluated during eligibility screening by a single evaluator, a Thai board-certified veterinary surgeon (MV). The evaluator had no access to the randomization schedules or the provisional hospital identification number–treatment code assignments. Therefore, radiographic eligibility assessments were performed without knowledge of the assigned or prospective treatment group.

Radiographic evidence of hip osteoarthritis was defined by the presence of degenerative changes affecting at least one hip joint, including osteophyte formation, subchondral sclerosis, remodeling of the femoral head or neck, acetabular changes, joint incongruity, or radiographic changes consistent with hip dysplasia-associated osteoarthritis. Radiographs were used to confirm study eligibility and were not used as an outcome measure, severity-stratification variable, or covariate in the efficacy analyses. Formal radiographic grading of hip dysplasia or osteoarthritis severity was not performed. Because radiographs were evaluated by a single evaluator for eligibility confirmation only, no consensus process among multiple evaluators was performed.

### 2.4. Severity Stratification and Randomization

Before treatment allocation, each dog underwent a standardized orthopedic assessment using the clinical scoring system adapted from Moreau et al. [6]. For randomization purposes, the articular pain score was assessed separately for each hip during palpation and manipulation. Dogs with pain detected on hip palpation or manipulation underwent radiographic examinations to confirm the presence of hip osteoarthritis. Dogs that met all eligibility criteria then underwent force-plate gait analysis to identify the index hindlimb.

For stratified randomization, a bilateral hip pain severity score was calculated as the sum of the articular pain scores from the left and right hips. Because the articular pain score for each hip ranged from 0 to 3, the bilateral hip pain severity score ranged from 0 to 6. Dogs with a score of 0 were not eligible for inclusion. Dogs with scores of 1–3 were classified as mild-to-moderate for randomization purposes, whereas dogs with scores of 4–6 were classified as severe. This bilateral hip pain severity score was used as a pragmatic pain-focused stratification variable and was distinct from the total OAS, which included lameness, bilateral articular mobility, and bilateral articular pain and was retained as a clinical outcome measure.

Separate randomization schedules were generated in advance for the mild-to-moderate and severe strata using Microsoft Excel 2019 version 16.0 (Microsoft Corporation, Redmond, WA, USA). Within each stratum, treatment codes were arranged in randomly permuted blocks of five, with each complete block containing one allocation to each of the five treatment groups. The schedules and treatment-code key were maintained exclusively by an independent investigator who was not involved in recruitment, eligibility assessment, clinical outcome evaluation, or statistical analysis.

Recruitment and follow-up assessments were conducted concurrently throughout the study period. Following preliminary screening and severity classification, the hospital identification numbers of prospective participants were provisionally entered into successive positions in the corresponding pre-generated randomization schedules according to recruitment order. These entries were made solely by the independent investigator before the scheduled pretreatment visits. At the pretreatment visit, eligibility and participation were confirmed, written informed consent was obtained from the owner, and the study medication corresponding to the reserved treatment code was dispensed. Clinical investigators responsible for recruitment and outcome assessment had no access to the randomization schedules or treatment-code key.

Some prospective participants whose hospital identification numbers had been provisionally entered into the schedules did not attend their scheduled pretreatment visits. These dogs did not provide informed consent or receive study medication. Their reserved positions remained unfilled, while recruitment continued using subsequent positions in the schedules, resulting in incomplete randomization blocks.

### 2.5. Blinding Procedures

A double-dummy procedure was used to maintain blinding because the study treatments were administered in both liquid and capsule formulations. The liquid placebo consisted of the meloxicam vehicle without an active pharmaceutical ingredient and was administered orally at a volume equivalent to the calculated meloxicam dose. The capsule placebo consisted of sunflower oil in a soft gelatin capsule that was externally indistinguishable from the active marine-derived lipid capsules and contained no marine-derived lipid extract. Dog owners and clinical investigators responsible for recruitment and outcome assessments remained blinded to treatment allocation. Study medications were prepared and dispensed by the independent investigator responsible for maintaining the randomization schedules, who was not involved in recruitment or clinical outcome evaluation. Statistical analyses were performed using numerical treatment codes (code 1–5), and the statistician was unaware of the corresponding treatment identities during the analyses.

### 2.6. Treatment Groups, Intervention Details, and Welfare Monitoring

#### 2.6.1. Study Products and Formulation Details

The study interventions included meloxicam oral suspension (Metacam^®^ 0.15%; Boehringer Ingelheim Vetmedica GmbH, Ingelheim am Rhein, Germany), PCSO-524 (Antinol^®^; Alpha Laboratories (NZ) Ltd., Auckland, New Zealand; batch no. AL46424), and EAB-277 (Antinol^®^ Rapid; New Zealand Health Manufacturing Ltd., Auckland, New Zealand; batch no. NHB0822). Study products were supplied by Pharmalink International Ltd. (Hong Kong SAR, China). PCSO-524 was supplied as a commercial stabilized marine lipid formulation derived from New Zealand green-lipped mussel (Perna canaliculus). EAB-277 was supplied as a commercial marine-derived lipid formulation containing New Zealand green-lipped mussel oil and high-phospholipid krill oil, with olive oil and capsule excipients. Available label-reported product composition in-formation for PCSO-524 and EAB-277 is provided in Supplementary Table S1. Study products were stored in their original packaging according to the supplier’s instructions until dispensing. Batch numbers were available for the marine-based fatty acid products, PCSO-524 and EAB-277, but were not available to the investigators for the meloxicam or placebo formulations. Batch-specific quantitative fatty acid profiles, certificates of analysis, and purified active-extract concentrations were not available to the investigators.

#### 2.6.2. Treatment Administration and Dosing

Dogs were allocated to one of five treatment groups for 4 weeks. All study treatments were administered orally once daily. For marine-based fatty acid products, the reported 5 mg/kg dose referred to the total commercial capsule formulation, not to purified active extract content. Capsule doses were calculated using each dog’s ideal body weight and rounded upward according to the capsule-dosing procedure. The actual administered dose ranges were 5.0–5.8 mg/kg for PCSO-524 and 5.0–5.43 mg/kg for EAB-277.

In the combination group, dogs received meloxicam at 0.2 mg/kg on the first day, followed by 0.1 mg/kg once daily, together with PCSO-524 at a target total formulation dose of 5 mg/kg once daily.

In the meloxicam group, dogs received meloxicam at 0.2 mg/kg on the first day, followed by 0.1 mg/kg once daily, together with the matching capsule placebo administered at a quantity equivalent to the calculated PCSO-524 dose.

In the placebo group, dogs received the liquid placebo at a volume equivalent to the calculated meloxicam dose, together with the matching capsule placebo administered at a quantity equivalent to the calculated PCSO-524 dose.

In the PCSO-524 group, dogs received PCSO-524 at a target total formulation dose of 5 mg/kg once daily, together with the liquid placebo administered at a volume equivalent to the calculated meloxicam dose.

In the EAB-277 group, dogs received EAB-277 at a target total formulation dose of 5 mg/kg once daily, together with the liquid placebo administered at a volume equivalent to the calculated meloxicam dose. EAB-277 was supplied in soft gelatin capsules that were externally indistinguishable from the PCSO-524 and capsule-placebo formulations.

#### 2.6.3. Adherence, Co-Interventions, and Concomitant Medications

Owners were asked to return all unused capsules and liquid study-medication bottles at each follow-up visit. Treatment adherence was assessed by capsule count, measurement of the remaining liquid volume in returned bottles, and owner interview. Owners were instructed to maintain the dog’s usual diet, routine daily activity, and lifestyle during the study period. They were asked not to introduce new rehabilitation therapy, structured exercise programs, nutraceutical supplements, analgesic medications, anti-inflammatory medications, or other interventions expected to affect osteoarthritis related pain or mobility. Concurrent medications or supplements related to osteoarthritis management were discontinued according to the study protocol before treatment initiation. At each follow-up visit, owners were interviewed to confirm adherence to these instructions and to identify any changes in diet, activity, medication, supplement use, rehabilitation, or other management.

#### 2.6.4. Welfare Monitoring and Rescue-Withdrawal Criterion

The approved study protocol included a predefined pain-related rescue-withdrawal criterion. Any dog whose mean CBPI Pain Interference Score increased by ≥3 points relative to the Day 0 score at an assessment was classified as a treatment failure, withdrawn from the study, and provided with appropriate clinical management as determined by the attending veterinarian. This criterion identified substantial deterioration in pain-related functional interference rather than an absolute level of acceptable pain.

Animal welfare monitoring included scheduled clinical reassessments at Weeks 0, 2, and 4, supplemented by owner observations and direct communication with the research team throughout the study. Owners were provided with telephone and project-specific instant messaging applications through which they could report concerns regarding pain, mobility, adverse events, or changes in their dogs’ general condition between scheduled visits.

Participation was voluntary, and owners retained the right to withdraw their dogs at any time without having to wait for the predefined rescue-withdrawal threshold to be reached. Dogs requiring clinical attention could be presented to the Veterinary Teaching Hospital for reassessment and appropriate treatment. Dogs could also be withdrawn if adverse events or intercurrent clinical conditions occurred and continued participation was considered inappropriate by the investigators or attending veterinarians.

### 2.7. Sample-Size Determination

The sample-size calculation was performed for the primary objective outcome, PVF of the index hindlimb. The calculation was conducted using G*Power version 3.1.9.4 (Franz Faul, Universität Kiel, Kiel, Germany), with the test family specified as F tests and the statistical test specified as ANCOVA: fixed effects, main effects, and interactions. The calculation used an a priori design with five treatment groups, one covariate representing baseline PVF, a two-sided type I error rate of 0.05, 80% statistical power, and a medium effect size of f = 0.25.

The effect size was selected as a pragmatic planning assumption for detecting a clinically relevant between-group difference in baseline-adjusted PVF. This assumption was informed by previous force-plate data from our group [20], in which a treatment group showed an approximately 4%BW increase in PVF over a 4-week treatment period. However, that previous study did not provide a direct active treatment versus placebo estimate for the present five-group design or a definitive estimate of between-group variability for the current trial. Therefore, the selected effect size should be interpreted as a planning assumption rather than a precisely estimated effect from prior placebo-controlled data.

Using these assumptions, the minimum required sample size was 196 dogs. This value was rounded upward to a planned total sample size of 200 dogs, corresponding to approximately 40 dogs per treatment group. The sample-size calculation followed an ANCOVA framework with baseline adjustment and was used as an approximation for the primary PVF analysis. A simulation-based sample-size calculation for the final repeated-measures linear mixed model was not performed. In the final analysis, post-treatment PVF at Weeks 2 and 4 was evaluated using a baseline-adjusted linear mixed model, with baseline PVF included as a covariate and repeated post-baseline measurements modeled within dog.

### 2.8. Outcome Assessments

Assessments were performed before treatment initiation at Week 0 and repeated at Weeks 2 and 4. The principal efficacy outcome was peak vertical force (PVF) of the index hindlimb measured by force-plate gait analysis. Additional efficacy outcomes included the veterinarian-assessed orthopedic assessment score (OAS), the owner-completed Liverpool Osteoarthritis in Dogs (LOAD) questionnaire, and the Canine Brief Pain Inventory (CBPI). The CBPI included two pain-related domains (pain severity and pain interference) and a separately evaluated single global quality-of-life (QOL) item.

Treatment safety and tolerability were evaluated using reported adverse events and hematological and serum biochemical variables.

#### 2.8.1. Peak Vertical Force

Peak vertical force was measured using two force plates (Model OR6-6; Advanced Mechanical Technology, Inc., Watertown, MA, USA) embedded in the center of a 10-m runway. Force-plate data were processed using Cortex software version 4.0 (Motion Analysis Corporation, Santa Rosa, CA, USA). Three timing sensors positioned at 2-m intervals were used to measure running velocity and acceleration. Dogs were required to run at a velocity of 1.8 to 2.2 m/s, with acceleration maintained between −0.5 and 0.5 m/s^2^ [20]. The same handler guided each dog across the runway throughout the study to reduce variation associated with handling.

Each trial was video recorded to verify complete paw contact with the force plate. A trial was considered valid when the paw made complete contact with the force plate and the prespecified velocity and acceleration criteria were met. Six valid trials were obtained for each dog at each assessment [20].

PVF was defined as the peak vertical force of the index hindlimb, normalized to body weight and expressed as percentage of body weight (%BW). For dogs with bilateral hip osteoarthritis, the painful hindlimb with the lower PVF at baseline was designated as the index hindlimb. This approach was used to identify the more functionally affected hindlimb at baseline and to maintain a consistent limb-specific PVF outcome for longitudinal analysis. The same index hindlimb was retained for analyses at Weeks 2 and 4, irrespective of subsequent changes in PVF in either hindlimb. The mean PVF obtained from the six valid trials for the index hindlimb was used for statistical analysis [20].

#### 2.8.2. Orthopedic Assessment Score

Orthopedic status was evaluated by a board-certified orthopedic surgeon (MV) using a clinical scoring system adapted from Moreau et al. [6]. The OAS included lameness, articular mobility, and articular pain components. Lameness was scored once per dog, whereas articular mobility and articular pain were scored separately for each hip. Hip joint pain was assessed during extension of the hip joint. The total OAS was calculated as the sum of the lameness score, left and right hip articular mobility scores, and left and right hip articular pain scores, giving a total possible score of 0 to 18. Higher scores indicated greater orthopedic impairment. The same scoring system was used at each assessment time point. The detailed scoring criteria are provided in Table 1.

#### 2.8.3. Owner-Reported Outcome Assessments

Owner-reported outcomes were assessed using Thai-language versions of the LOAD and CBPI questionnaires. The questionnaires were translated into Thai by a bilingual university-based expert and reviewed by three Thai clinicians for clarity, clinical appropriateness, and owner comprehensibility. The Thai-language CBPI version had been used previously in a published clinical trial of canine osteoarthritis, whereas the Thai-language LOAD version was translated and clinically reviewed for use in the present study but has not been formally validated as a Thai-language instrument. Questionnaires were administered in printed paper format at each assessment time point. Owners were instructed and agreed that the same owner or primary caregiver should complete the questionnaires at baseline, Week 2, and Week 4 whenever possible.

Owners completed the LOAD questionnaire to assess mobility and functional ability associated with osteoarthritis [7,8,9]. The questionnaire consisted of two sections: the first addressed the dog’s general exercise behavior, and the second assessed mobility during routine daily activities. The scored section contained 13 items, each with five response categories. Responses were converted to ordinal scores, and the total LOAD score was used for statistical analysis. Higher LOAD scores indicated poorer mobility and functional ability.

Owners also completed the Canine Brief Pain Inventory (CBPI), which comprises two pain-related domains and a single quality-of-life (QOL) item [10]. The pain severity domain assesses pain intensity, whereas the pain interference domain evaluates the effects of pain on daily activities. Pain severity and pain interference scores were calculated as the means of their respective items, with higher scores indicating greater pain severity or pain-related interference.

The original CBPI quality-of-life item uses a five-point categorical scale ranging from 1 (poor) to 5 (excellent), with higher scores indicating better quality of life. In the present study, this item was intentionally reverse-coded and recorded in the study database as Inverse QOL, using the transformation: Inverse QOL = 6 − original QOL score. The reverse-coded categories were 1 = excellent, 2 = very good, 3 = good, 4 = fair, and 5 = poor. This variable was reported as the quality-of-life impairment score, with higher values indicating poorer quality of life and lower values indicating better quality of life. The quality-of-life item was analyzed separately and did not contribute to the CBPI pain severity or pain interference scores.

Therefore, for all LOAD and CBPI outcomes reported in this study, lower scores represented improvement.

#### 2.8.4. Safety Assessment

A 3-mL blood sample was collected from the cephalic vein of each dog at each assessment. One milliliter was transferred into a tube containing ethylenediaminetetraacetic acid for a complete blood count, and the remaining 2 mL was placed in a serum collection tube for serum biochemical analysis.

Hematological analyses were performed using an automated veterinary hematology analyzer (XN-1000Vet, Sysmex Corporation, Kobe, Japan). Serum biochemical analyses were performed using an automated clinical chemistry analyzer (ILab Taurus analyzer, Instrumentation Laboratory, Milan, Italy).

Hematological variables included packed cell volume (PCV), white blood cell (WBC) count, and platelet count. Serum biochemical variables included blood urea nitrogen (BUN), creatinine, alanine aminotransferase (ALT), alkaline phosphatase (ALP), total protein (TP), and albumin (ALB).

Reported adverse events, treatment discontinuations, and withdrawals were recorded throughout the study.

### 2.9. Statistical Analysis

Statistical analyses were performed using IBM SPSS Statistics for Windows, version 31.0 (IBM Corp., Armonk, NY, USA). All statistical tests were two-sided, and statistical significance was set at *p* < 0.05 unless otherwise specified. Continuous variables were summarized as mean ± SD when approximately normally distributed. Ordinal or markedly skewed variables were summarized as median [interquartile range]. Categorical variables were summarized as counts and percentages. Where applicable, pairwise comparisons were adjusted within each outcome using the Bonferroni method. However, no formal study-wide adjustment was applied across all outcome measures. This decision reflected the multidimensional design of the study, in which PVF, OAS, LOAD, CBPI, and laboratory variables were intended to capture distinct aspects of treatment response rather than repeated tests of a single construct. Accordingly, findings from secondary and exploratory outcomes were interpreted cautiously, with attention to effect estimates, 95% confidence intervals, adjusted *p*-values where applicable, consistency across measures, direction of change, and clinical relevance rather than statistical significance alone.

#### 2.9.1. Analysis Populations and Missing Data

The efficacy analysis included randomized dogs with baseline data and at least one post-baseline efficacy assessment. The safety analysis included all randomized dogs that received at least one dose of the assigned study treatment. Dogs were analyzed according to their randomized treatment group.

The efficacy analysis population was considered a modified intention-to-treat population with available-case analysis. All available observations were included in the analyses. Missing post-baseline measurements were not replaced by last-observation-carried-forward, next-observation-carried-backward, group-mean substitution, or other single-value imputation methods. Reasons for withdrawal or missing data were summarized by treatment group when available.

#### 2.9.2. Baseline Characteristics

Baseline demographic and clinical characteristics were summarized by treatment group to evaluate comparability among treatment groups. Continuous baseline variables were compared among treatment groups using one-way ANOVA or Kruskal-Wallis tests, as appropriate. Categorical variables were compared using chi-square tests or Fisher’s exact tests, as appropriate. These analyses were used to describe baseline comparability and were not used as the sole basis for determining covariate adjustment in post-treatment analyses.

#### 2.9.3. Primary Analysis: Baseline-Adjusted Linear Mixed Model for PVF

A baseline-adjusted linear mixed model was used to evaluate post-treatment PVF at Weeks 2 and 4. This analysis followed an ANCOVA framework in which baseline PVF was included as a covariate rather than analyzed as a repeated outcome. Post-treatment PVF at Weeks 2 and 4 was specified as the dependent variable. Treatment group, week, and the treatment group-by-week interaction were included as fixed effects, with baseline PVF included as a covariate. Dog identification was specified as the repeated subject, and within-dog correlations between repeated post-baseline measurements were modeled using a first-order autoregressive covariance structure.

Week 4, the end of the treatment period, was defined as the primary post-treatment assessment time for the PVF outcome. Estimated marginal means were calculated for each treatment group at Weeks 2 and 4. Overall fixed effects of treatment group, week, treatment group-by-week interaction, and baseline PVF were evaluated. At Week 4, baseline-adjusted between-group comparisons were performed to address the primary study objectives. These included comparisons between each active treatment group and placebo, and direct comparisons between the combination treatment and its individual components, meloxicam and PCSO-524. Pairwise estimated marginal mean comparisons were adjusted using the Bonferroni method.

Within-group comparisons between Week 2 and Week 4 were also performed using model-based pairwise comparisons with Bonferroni adjustment. Because baseline PVF was included as a covariate, baseline-to-Week 2 and baseline-to-Week 4 changes were summarized descriptively rather than tested within this model. Observed PVF values and descriptive changes from baseline to Weeks 2 and 4 were reported by treatment group. Model assumptions were assessed using residual diagnostic plots.

#### 2.9.4. Secondary Analysis: PVF Responder Analysis

PVF responder analysis was performed as a secondary analysis to provide an individual-dog perspective on objective limb-loading improvement. PVF gain was calculated as the absolute change in PVF of the index hindlimb from baseline to each post-treatment assessment: PVF gain at Week 2 = PVF at Week 2 minus PVF at Week 0, and PVF gain at Week 4 = PVF at Week 4 minus PVF at Week 0. All PVF gain values were expressed as %BW. Week 4 was considered the primary post-treatment time point for interpretation of responder findings, whereas Week 2 responder results were considered secondary descriptive analyses.

During the statistical analysis stage, an absolute increase in PVF of >5%BW from baseline was selected as the main responder threshold, based on published recommendations for interpreting ground reaction force changes in dogs [12]. This threshold was used as a conservative individual-dog improvement criterion for objective limb-loading response, informed by published recommendations regarding interpretation of ground reaction force changes and measurement variability in dogs [12]. It was not derived from a study-specific test-retest measurement-error estimate and was not intended to represent a formally validated minimal clinically important difference. Because the cited recommendations were published after recruitment had ended, this threshold was considered an analysis-stage responder definition rather than a protocol-specified criterion established before recruitment. Dogs with a PVF gain of >5%BW were classified as responders, whereas dogs with a PVF gain of ≤5%BW were classified as non-responders.

An additional responder analysis using a lower PVF improvement threshold of ≥3.5%BW from baseline was performed as an exploratory sensitivity analysis [12]. This lower threshold was used to examine whether treatment-placebo patterns were consistent when a more sensitive responder definition was applied. Dogs with a PVF gain of ≥3.5%BW were classified as responders for this exploratory analysis. Separate responder variables were created for Weeks 2 and 4 for each threshold.

Relative risks for PVF response were estimated using modified Poisson regression with a log link and robust covariance estimation. The placebo group was used as the reference group. Relative risks, robust 95% confidence intervals, and *p*-values were reported for each active-treatment group compared with placebo. Responder counts and percentages were also summarized by treatment group.

No hierarchical confirmatory testing framework was applied, and no formal multiplicity adjustment was performed across responder thresholds, assessment time points, or treatment comparisons. Therefore, responder *p*-values were interpreted as nominal, and responder findings were considered supportive or exploratory rather than confirmatory evidence of treatment efficacy.

#### 2.9.5. Score-Based Clinical Outcomes

Score-based clinical outcomes included veterinarian-assessed OAS and owner-reported LOAD and CBPI measures. The CBPI outcomes comprised pain severity and pain interference domain scores and the separately analyzed reverse-coded global QOL item (QOL impairment score). These outcomes were summarized as median [interquartile range] and analyzed using nonparametric methods. The Friedman tests were based on complete cases with observations at all three assessment times, whereas descriptive statistics were calculated using available observations at each visit.

Within-group changes across baseline, Week 2, and Week 4 were evaluated using Friedman tests. When the overall Friedman test was significant, pairwise comparisons between time points were performed with Bonferroni adjustment. Within-group analyses were used to describe temporal changes within each treatment group and were not interpreted as evidence of treatment efficacy compared with placebo.

Cross-sectional between-group comparisons of score-based outcomes were performed separately at baseline, Week 2, and Week 4 using independent-samples Kruskal-Wallis tests. When the overall Kruskal-Wallis test was significant, pairwise comparisons between treatment groups were performed with Bonferroni adjustment. Comparative interpretation of score-based outcomes was based primarily on between-group analyses, with attention to whether significant within-group changes were accompanied by corresponding between-group differences.

Additional exploratory change-from-baseline analyses were performed for owner-reported LOAD and CBPI outcomes to compare score changes among treatment groups. For each dog, change from baseline was calculated as the post-treatment score minus the baseline score at Week 2 and Week 4. Negative values therefore indicated improvement for LOAD and CBPI outcomes. Because the change-from-baseline values were not normally distributed, between-group comparisons of change scores were performed using independent-samples Kruskal-Wallis tests. These analyses were considered exploratory and were used to evaluate whether within-group improvements were supported by between-group differences in score changes.

#### 2.9.6. Laboratory Safety Variables

Hematological and serum biochemical variables were summarized descriptively by treatment group and assessment time. Packed cell volume, platelet count, WBC count, blood urea nitrogen, serum creatinine, total protein, and albumin were analyzed using linear mixed models for repeated measures. Each model included treatment group, week, and the treatment group-by-week interaction as fixed effects, with dog identification specified as the repeated subject. Within-group comparisons over time and between-group comparisons at individual time points were performed with Bonferroni adjustment where applicable.

Because alanine aminotransferase and alkaline phosphatase activities were markedly skewed, these variables were summarized as median [Q1–Q3] and analyzed using nonparametric methods. Within-group changes over time were evaluated using Friedman tests, with Bonferroni-adjusted pairwise comparisons where applicable. Cross-sectional between-group comparisons at baseline, Week 2, and Week 4 were performed using Kruskal-Wallis tests, with Bonferroni-adjusted pairwise comparisons where applicable.

Laboratory abnormalities were interpreted together with clinical adverse events, treatment discontinuations, withdrawals, and clinical relevance.

#### 2.9.7. Adverse Events

Adverse events were summarized in the safety population. Events were described by treatment group, time of onset, clinical category, severity, action taken, outcome, and whether the event led to treatment discontinuation or withdrawal from the study. No adverse event was classified as treatment-related unless a causal relationship to the assigned treatment was considered at least possible by the investigators.

## 3. Results

### 3.1. Study Population and Baseline Characteristics

This clinical trial was conducted in client-owned dogs with hip osteoarthritis presented to the Kasetsart University Veterinary Teaching Hospital, Bang Khen Campus, between January 2019 and August 2021. A total of 313 dogs were assessed for eligibility, of which 203 were enrolled following written owner consent and received study treatment in one of the five treatment groups. The enrolled and treated population comprised 79 females and 124 males, with a mean age of 60.55 ± 29.52 months and a mean body weight of 34.46 ± 7.24 kg.

Ten breeds were represented, most commonly Golden Retriever (*n* = 96), Labrador Retriever (*n* = 42), and Siberian Husky (*n* = 32). Other breeds included mixed-breed dogs (*n* = 8), Alaskan Malamute (*n* = 7), American Pit Bull Terrier (*n* = 6), Rottweiler (*n* = 5), German Shepherd Dog (*n* = 3), Thai Bangkaew Dog (*n* = 3), and Thai Ridgeback Dog (*n* = 1). Based on the five-point body condition scoring system, 84 dogs had a body condition score (BCS) of 3/5, 85 had a BCS of 4/5, and 34 had a BCS of 5/5.

**Figure 1:**
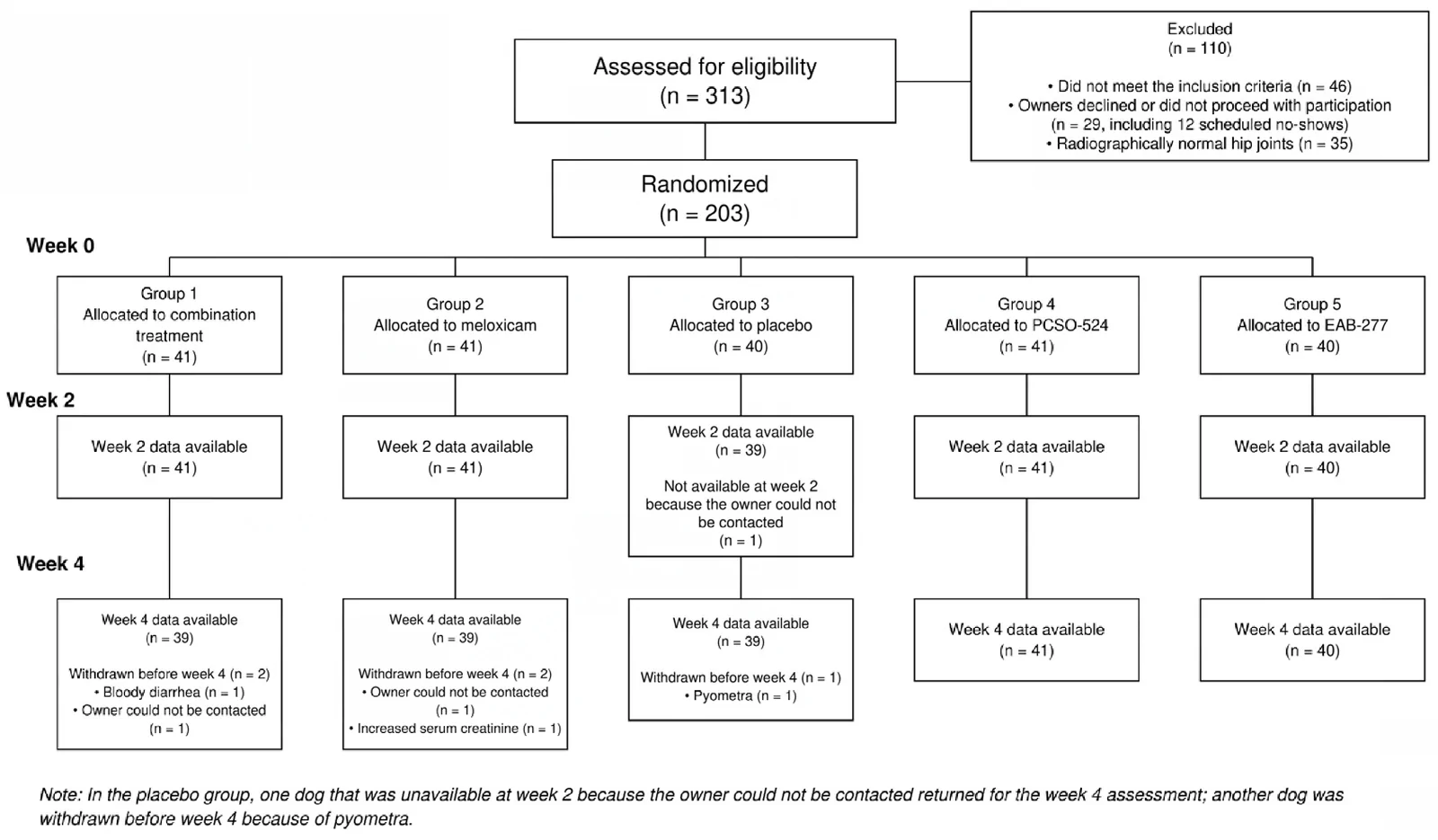
Flow diagram of dog screening, randomization, allocation, follow-up, withdrawal, and analysis.

Of the 110 dogs who were not enrolled, 46 did not meet the inclusion criteria, 35 had radiographically normal hip joints, and 29 did not proceed with participation. The latter group included 12 dogs whose hospital identification numbers had been provisionally entered into the randomization schedules, but who did not attend their scheduled pretreatment visits. These 12 dogs did not provide informed consent or receive study medication. One dog in the placebo group was unavailable for the Week 2 assessment because the owner could not be contacted but returned for the Week 4 assessment. Five dogs did not complete the Week 4 assessment: two in the combination group because of bloody diarrhea (*n* = 1) and inability to contact the owner (*n* = 1), two in the meloxicam group because of inability to contact the owner (*n* = 1) and increased serum creatinine concentration (*n* = 1), and one in the placebo group because of pyometra. Participant flow through the study is shown in Figure 1.

The baseline demographic and clinical characteristics of dogs with hip osteoarthritis are summarized by treatment group in Table 2. No significant differences were detected among treatment groups for any baseline demographic or clinical characteristic. Baseline severity classification was balanced among treatment groups. The proportions of dogs classified as mild-to-moderate and severe did not differ significantly among groups (*p* = 0.889).

Reconciliation of the original severity-specific randomization records identified 23 used blocks in the mild-to-moderate stratum, comprising 115 positions, of which eight remained unfilled. In the severe stratum, 20 blocks were used, comprising 100 positions, of which four remained unfilled. The remaining 107 and 96 documented allocations, respectively, corresponded to 203 enrolled and treated dogs with distinct hospital identification numbers. The treatment-group distributions were fully consistent with those reported in Table 2: 41 dogs in the combination group, 41 in the meloxicam group, 40 in the placebo group, 41 in the PCSO-524 group, and 40 in the EAB-277 group.

### 3.2. Objective Force Plate Assessment: Peak Vertical Force

#### 3.2.1. Baseline PVF and Baseline-Adjusted Linear Mixed Model Analysis

Baseline PVF was a significant covariate in the model (F = 900.672, *p* < 0.001). After adjustment for baseline PVF, there was no significant overall effect of treatment group (F = 1.292, *p* = 0.275), no significant effect of week (F = 3.225, *p* = 0.074), and no significant treatment group-by-week interaction (F = 2.031, *p* = 0.092). These findings indicate that baseline-adjusted post-treatment PVF did not differ significantly among treatment groups over the 4-week treatment period. As an exploratory sensitivity analysis, severity classification was added to the baseline-adjusted PVF mixed model together with the treatment group-by-severity interaction. In this exploratory model, severity classification was not significantly associated with post-treatment PVF (F = 0.047, *p* = 0.828), and no treatment group-by-severity interaction was detected (F = 0.960, *p* = 0.431). These findings did not change the interpretation of the primary PVF analysis.

Week 4 was defined as the primary post-treatment assessment time for the PVF outcome. At Week 4, there were no statistically significant baseline-adjusted differ-ences between any active treatment group and placebo after Bonferroni adjustment. Direct comparisons between the combination treatment and its individual compo-nents also did not demonstrate statistically significant differences. The adjusted mean differences, 95% confidence intervals, and Bonferroni-adjusted *p*-values for these question-directed comparisons are provided in Supplementary Table S2. Therefore, the primary baseline-adjusted mean PVF analysis failed to demonstrate superiority of any active treatment over placebo and failed to demonstrate additional benefit of the combination treatment over meloxicam or PCSO-524 alone.

**Figure 2:**
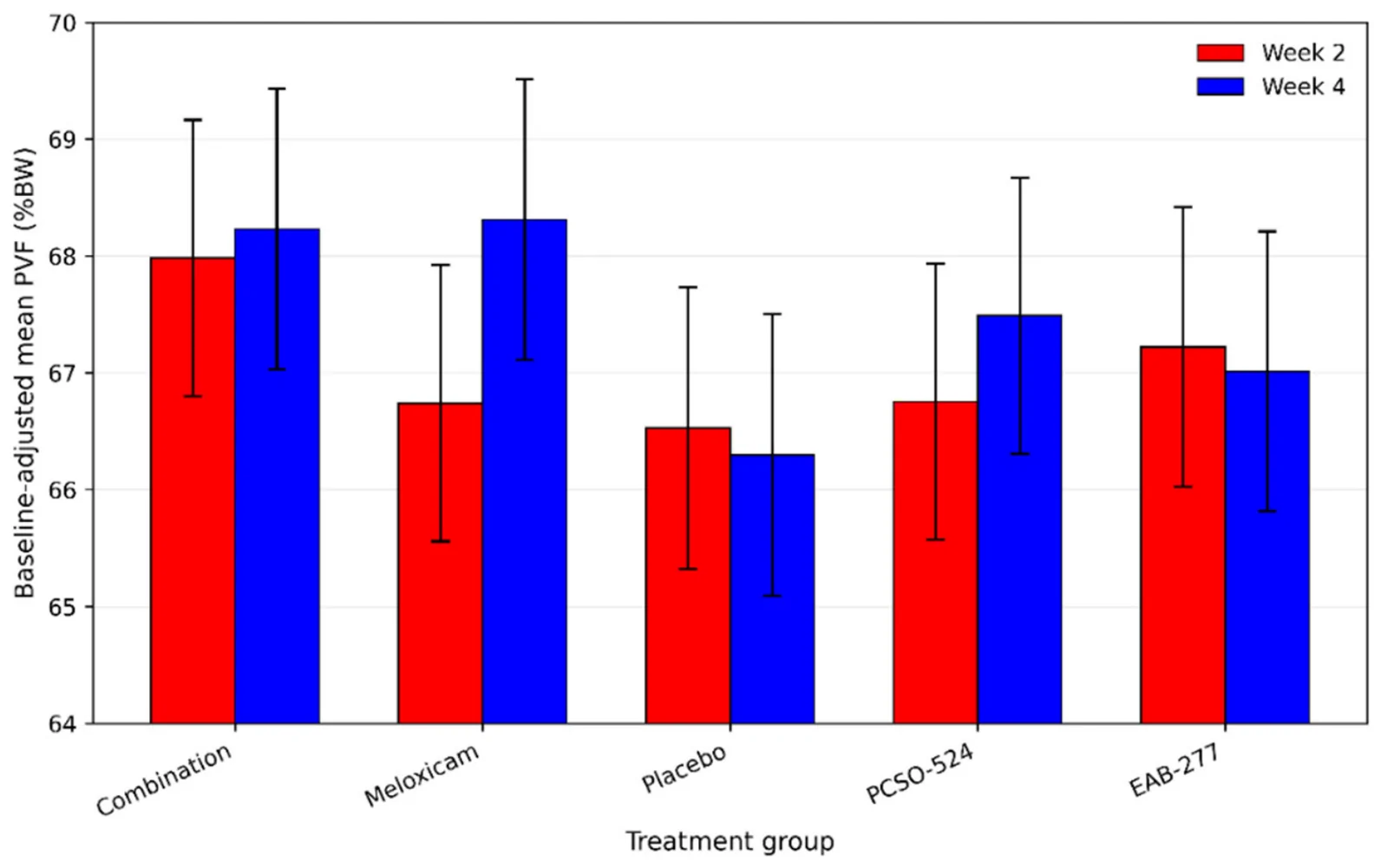
Baseline-adjusted estimated marginal means of PVF at Weeks 2 and 4 within each treatment group. Error bars represent 95% confidence intervals. Estimated marginal means were adjusted for baseline PVF, with the covariate evaluated at 65.54%BW. PVF = peak vertical force of the index hindlimb; %BW = percentage of body weight.

Baseline-adjusted estimated marginal means of PVF at Weeks 2 and 4 are shown in Figure 2 and Table 3. Within-group model-based pairwise comparisons showed that PVF increased significantly from Week 2 to Week 4 in the meloxicam group (mean difference = 1.57%BW; 95% CI, 0.53 to 2.62; Bonferroni-adjusted *p* = 0.003). No significant Week 2 to Week 4 differences were detected within the combination, placebo, PCSO-524, or EAB-277 groups. However, because the overall treatment group-by-week interaction was not statistically significant, this within-group finding was interpreted as supportive rather than evidence of treatment superiority.

Observed PVF values and paired individual descriptive changes from baseline to Weeks 2 and 4 are provided in Supplementary Table S3. These descriptive changes were included to aid clinical interpretation, whereas inferential comparisons of mean PVF were based on the baseline-adjusted linear mixed model. Because the primary baseline-adjusted mean PVF analysis failed to demonstrate treatment superiority, PVF responder analyses are reported separately and interpreted as supportive secondary individual-dog analyses rather than confirmatory evidence of overall treatment efficacy.

#### 3.2.2. Responder Analysis of PVF Improvement at Weeks 2 and 4

PVF responder analyses were performed as secondary analyses to provide an individual-dog perspective on objective limb-loading improvement. Results for the conservative >5%BW responder threshold are summarized in Table 4. At Week 2, no active treatment group showed a nominally significant higher responder probability compared with placebo.

At Week 4, using the conservative >5%BW threshold, the proportions of responders were 10/39 dogs (25.6%) in the combination group, 12/39 dogs (30.8%) in the meloxicam group, 3/39 dogs (7.7%) in the placebo group, 6/41 dogs (14.6%) in the PCSO-524 group, and 8/40 dogs (20.0%) in the EAB-277 group. Compared with placebo, meloxicam-treated dogs had a nominally higher responder probability at Week 4 (RR = 4.00; 95% CI, 1.22 to 13.08; *p* = 0.022). The combination group did not reach nominal statistical significance (RR = 3.33; 95% CI, 0.99 to 11.20; *p* = 0.051), and PCSO-524 and EAB-277 did not differ significantly from placebo using this threshold. Because responder analyses were secondary and no formal multiplicity adjustment was applied across responder thresholds, assessment time points, or treatment comparisons, these findings were interpreted as supportive rather than confirmatory.

An exploratory responder analysis using a lower threshold of ≥3.5%BW was also performed, and detailed results are provided in Supplementary Table S4. At Week 4, meloxicam and PCSO-524 showed nominal treatment-placebo differences using this exploratory threshold. However, the overall treatment-group test for the exploratory ≥3.5%BW threshold was not statistically significant, and no formal multiplicity adjustment was applied across responder thresholds or treatment comparisons. Therefore, findings based on the ≥3.5%BW threshold were interpreted as exploratory only.

### 3.3. Score-Based Clinical Outcomes

#### 3.3.1. Within-Group Changes in Score-Based Outcomes

Score-based outcomes were analyzed descriptively using within-group changes over time and comparative between-group analyses at each time point. Within-group improvements were interpreted as temporal changes within a treatment group and were not considered evidence of treatment superiority over placebo unless supported by corresponding between-group differences.

#### 3.3.2. Orthopedic Assessment Score

Orthopedic status was evaluated by a board-certified orthopedic surgeon who was blinded to treatment allocation. No significant within-group change in orthopedic assessment score (OAS) was detected across baseline, Week 2, and Week 4 in any treatment group. Friedman test *p*-values were 0.266 for the combination group, 0.596 for the meloxicam group, 0.093 for the placebo group, 0.224 for the PCSO-524 group, and 0.703 for the EAB-277 group.

#### 3.3.3. Owner Assessment: Liverpool Osteoarthritis in Dogs Questionnaire

The Liverpool Osteoarthritis in Dogs questionnaire (LOAD) was used as an owner-based assessment of mobility and functional performance. Within-group Friedman tests showed significant changes in LOAD score over time in the combination group (*p* = 0.016), PCSO-524 group (*p* = 0.005), and EAB-277 group (*p* = 0.038), but not in the meloxicam group (*p* = 0.097) or placebo group (*p* = 0.051). After Bonferroni correction, LOAD score was significantly reduced from baseline to Week 4 in the combination group (adjusted *p* = 0.013). In the PCSO-524 group, LOAD score was significantly reduced from baseline at both Week 2 (adjusted *p* = 0.018) and Week 4 (adjusted *p* = 0.023). Although the EAB-277 group showed a significant overall Friedman test, no pairwise comparison remained significant after Bonferroni correction (Table 5).

#### 3.3.4. Owner Assessment: Canine Brief Pain Inventory Score

The Canine Brief Pain Inventory (CBPI) was used to evaluate owner-assessed pain severity, pain-related interference with daily activities, and quality-of-life impairment. For the CBPI pain severity domain, significant overall within-group changes over time were detected in the PCSO-524 group (*p* = 0.014) and EAB-277 group (*p* = 0.039), but no Bonferroni-adjusted pairwise comparison remained statistically significant in any treatment group.

For the CBPI pain interference domain, significant within-group changes over time were detected in the combination group (*p* = 0.004), meloxicam group (*p* = 0.032), and EAB-277 group (*p* = 0.008), but not in the placebo group (*p* = 0.149) or PCSO-524 group (*p* = 0.211). After Bonferroni correction, the combination group showed a significant reduction in CBPI pain interference score from baseline to Week 4 (adjusted *p* = 0.009) and from Week 2 to Week 4 (adjusted *p* = 0.029). The EAB-277 group also showed a significant reduction from baseline to Week 4 (adjusted *p* = 0.024). Although the meloxicam group showed a significant overall Friedman test, none of the pairwise comparisons remained statistically significant after Bonferroni correction.

For the reverse-coded CBPI quality-of-life item (QOL impairment score), a significant overall within-group change over time was detected only in the combination group (Friedman test, *p* = 0.009). However, none of the pairwise comparisons remained statistically significant after Bonferroni adjustment. Therefore, no specific pairwise improvement in the QOL impairment score was established. The descriptive statistics and within-group comparisons for the CBPI measures are presented in Table 6.

Because these within-group findings did not directly test active treatment effects against placebo, they were interpreted cautiously and were considered supportive only when consistent with between-group comparisons and the primary PVF analysis.

#### 3.3.5. Cross-Sectional Between-Group Comparisons of Score-Based Outcomes

At baseline, no significant between-group differences were detected for any score-based outcome, supporting baseline comparability among treatment groups. At Week 2, the CBPI pain severity score differed significantly among treatment groups (*p* = 0.002). However, after Bonferroni adjustment, no pairwise comparison between treatment groups remained statistically significant. Therefore, this finding was interpreted as an overall week-2 between-group difference without a clearly identifiable pairwise treatment contrast. No significant between-group differences were detected for any score-based outcome at Week 4.

Additional exploratory change-from-baseline analyses were performed for owner-reported LOAD and CBPI outcomes. No significant between-group differences in change from baseline were detected for LOAD at Week 2 (*p* = 0.108) or Week 4 (*p* = 0.800), CBPI pain severity at Week 2 (*p* = 0.098) or Week 4 (*p* = 0.558), CBPI pain interference at Week 2 (*p* = 0.442) or Week 4 (*p* = 0.529), or reverse-coded CBPI quality-of-life impairment score at Week 2 (*p* = 0.725) or Week 4 (*p* = 0.132). These findings indicate that within-group improvements in owner-reported outcomes were not consistently supported by between-group differences in change from baseline.

### 3.4. Safety Assessment

Hematological and serum biochemical variables were evaluated as safety outcomes. Packed cell volume, platelet count, serum creatinine concentration, and serum albumin concentration showed no significant treatment effect, week effect, or treatment-by-week interaction. At the group level, serum creatinine concentration did not show a significant treatment effect, week effect, or treatment-by-week interaction, although one dog in the meloxicam group was withdrawn because of increased serum creatinine concentration identified at Week 4.

WBC count showed a significant treatment-by-week interaction (*p* = 0.028), but no significant overall treatment effect or week effect. A single between-group difference was detected at Week 4, with higher WBC count in the meloxicam group than in the EAB-277 group after Bonferroni adjustment (*p* = 0.036). However, no significant within-group changes over time were observed. BUN showed no significant treatment effect, week effect, or treatment-by-week interaction. Although BUN increased from baseline to Week 4 in the meloxicam group after Bonferroni adjustment (*p* = 0.034), no significant between-group differences were detected at any time point. Total protein showed a significant treatment-by-week interaction, with small transient changes in the meloxicam and PCSO-524 groups, but these changes were not accompanied by significant albumin changes or consistent between-group differences.

Because ALT and ALP activities were markedly skewed, these variables were summarized as median [Q1–Q3] and analyzed using nonparametric methods. No significant between-group differences in ALT activity were detected at baseline (*p* = 0.322), Week 2 (*p* = 0.382), or Week 4 (*p* = 0.076). Friedman tests showed a significant within-group change in ALT activity only in the combination group (*p* = 0.034), driven by a decrease from baseline to Week 4 after Bonferroni adjustment (*p* = 0.033). No significant within-group increases in ALT activity were detected in any treatment group. No significant between-group differences in ALP activity were detected at baseline (*p* = 0.858), Week 2 (*p* = 0.783), or Week 4 (*p* = 0.844), and Friedman tests showed no significant within-group changes in ALP activity over time in any treatment group. Overall, no evidence of treatment-associated increases in ALT or ALP activity was detected over the 4-week study period.

Overall, no consistent group-level safety signal was detected in hematological or serum biochemical variables over the 4-week study period. These findings were interpreted as short-term laboratory safety and tolerability observations rather than evidence of safety for chronic treatment.

### 3.5. Adverse Events and Withdrawals

All randomized dogs were included in the safety assessment. Follow-up data were available for all dogs at Week 2, except for one dog in the placebo group whose owner could not be contacted; this dog returned for the Week 4 assessment. At Week 4, outcome data were unavailable for two dogs in the combination group, two dogs in the meloxicam group, and one dog in the placebo group.

Three dogs were withdrawn because of adverse events or intercurrent clinical conditions during the study period. In the combination group, one dog developed bloody diarrhea at the beginning of Week 4 and was withdrawn from the study. The dog was managed with gastrointestinal treatment and temporary discontinuation of the study products, and the clinical signs resolved within two days. This event was considered possibly related to the study intervention. One dog in the combination group was lost to follow-up because the owner could not be contacted.

In the meloxicam group, one dog showed increased serum creatinine concentration at Week 4 and was withdrawn from the study. The study medication was discontinued, and the dog received fluid therapy with continued monitoring of serum biochemical variables and systemic clinical signs. Serum creatinine returned to the reference interval after two weeks of monitoring. After fluid therapy was discontinued, serum creatinine remained within the reference interval without clinical signs for at least two months. This event was considered possibly related to the study intervention. One dog in the meloxicam group was lost to follow-up because the owner could not be contacted.

In the placebo group, one dog developed pyometra at Week 4 and was withdrawn from the study. The dog was managed by ovariohysterectomy and supportive care and fully recovered. This condition was considered unrelated to the study intervention. No dogs in the PCSO-524 or EAB-277 groups were withdrawn or lost to follow-up.

No consistent pattern of adverse events suggesting a group-level treatment-related safety signal was identified. No pain-related rescue withdrawals were recorded under the predefined criterion of a ≥3-point increase in mean CBPI Pain Interference Score relative to Day 0. Consequently, no withdrawal was attributed to treatment failure as defined by this criterion, and no rescue analgesic intervention affected subsequent efficacy assessments or analyses.

## 4. Discussion

This randomized, double-blind, placebo-controlled trial evaluated short-term objective limb-loading response, owner-reported functional outcomes, and laboratory safety findings in client-owned dogs with naturally occurring hip osteoarthritis treated with meloxicam, marine-based fatty acid compounds, combination treatment, or placebo. The primary baseline-adjusted mean PVF analysis failed to demonstrate superiority of any active treatment over placebo over the 4-week treatment period, and this primary result governs the overall interpretation of the study. In the secondary PVF responder analysis, meloxicam showed a nominal Week 4 treatment-placebo responder signal using the conservative analysis-stage >5%BW threshold; however, this finding was interpreted as supportive rather than confirmatory and should not supersede the primary mean PVF result. Because no hierarchical confirmatory testing framework or formal multiplicity adjustment was applied across responder thresholds, assessment time points, or treatment comparisons, the responder findings were interpreted cautiously. Owner-reported outcomes showed a different pattern: combination treatment was associated with improvements in LOAD and CBPI pain interference, PCSO-524 showed within-group improvement mainly in LOAD score, and EAB-277 showed within-group improvement in CBPI pain interference. These owner-reported findings should be interpreted as descriptive or supportive secondary findings rather than evidence of treatment superiority, because most were based on within-group changes over time and were not consistently accompanied by significant between-group differences versus placebo or by demonstrated superiority in the primary baseline-adjusted mean PVF analysis. Together, these findings indicate that objective gait analysis and owner-reported outcomes provide complementary, but not interchangeable, information when evaluating treatment response in dogs with hip osteoarthritis.

The baseline-adjusted linear mixed model was used to evaluate mean post-treatment PVF while accounting for baseline PVF. This analysis did not show a significant overall treatment effect, week effect, or treatment group-by-week interaction. Therefore, the primary baseline-adjusted mean PVF analysis failed to demonstrate superiority of any active treatment over placebo over the 4-week treatment period. In addition, direct Week 4 comparisons did not demonstrate additional benefit of the meloxicam and PCSO-524 combination compared with either meloxicam or PCSO-524 alone. These findings should not be interpreted as evidence of equivalence among treatments or as proof of absence of clinically relevant effects. Rather, under the conditions of the present study, the primary mean PVF analysis did not provide sufficient evidence to demonstrate treatment superiority or additional benefit of the combination treatment.

Responder analysis was used to provide an additional individual-dog perspective on objective limb-loading improvement. The main responder threshold defined response as an absolute increase in PVF of >5%BW from baseline. This threshold was selected during the statistical analysis stage as a conservative PVF-based criterion informed by published recommendations for interpreting ground reaction force changes in dogs with osteoarthritis. The threshold was intended to reduce the likelihood that small changes related to measurement variability would be classified as response, but it was not derived from a study-specific test-retest measurement-error estimate. It was not intended to represent a formally validated minimal clinically important difference. At Week 4, meloxicam-treated dogs were nominally more likely than placebo-treated dogs to achieve PVF responder status using the conservative >5%BW threshold. However, because the responder analysis was secondary and no hierarchical confirmatory testing framework or formal multiplicity adjustment was applied across responder thresholds, assessment time points, or treatment comparisons, this finding should be interpreted as supportive rather than confirmatory.

An exploratory responder analysis using a lower threshold of ≥3.5%BW was also performed as a more sensitive PVF responder definition. This lower threshold identified more responders than the conservative >5%BW threshold. At Week 4, meloxicam and PCSO-524 showed nominally higher responder probabilities than placebo using this exploratory threshold; however, the overall treatment-group effect was not significant. Therefore, the ≥3.5%BW findings were considered exploratory and were interpreted cautiously. Because the primary baseline-adjusted mean PVF analysis failed to demonstrate a significant treatment effect or treatment group-by-week interaction, the responder findings should be interpreted as supportive evidence of short-term objective improvement at the individual-dog level rather than definitive evidence of overall treatment superiority.

The apparent difference between the mean-based PVF analysis and the responder analysis is clinically important. Mean group comparisons estimate the average treatment effect across all dogs, whereas responder analysis evaluates whether individual dogs achieve a defined threshold of objective limb-loading improvement. Dogs with naturally occurring hip osteoarthritis are clinically heterogeneous, with variation in disease severity, chronicity, pain sensitivity, compensatory gait patterns, body condition, activity level, and structural joint changes [27,28,29,30]. The multidimensional nature of OA pain further complicates the assessment and interpretation of treatment outcomes [31]. Such heterogeneity may increase variability and reduce the ability to detect an average treatment effect, even when a subset of dogs achieves an individual-dog objective limb-loading response. This may explain why the baseline-adjusted mean PVF analysis failed to demonstrate treatment superiority, whereas the secondary responder analysis identified a nominal Week 4 meloxicam responder signal using the conservative >5%BW threshold. Therefore, the PVF responder finding should be interpreted as supportive evidence of short-term objective response in a subset of dogs, rather than confirmatory evidence of overall treatment superiority.

Clinical signs and ground reaction force measurements in dogs with osteoarthritis may also wax and wane over short periods [12,32]. Therefore, PVF measured at Week 2 or Week 4 represents objective limb-loading status at that specific visit and may not fully capture sustained treatment response. This issue supports cautious interpretation of responder status at a single post-treatment time point, particularly because responder analyses were secondary and were not part of a hierarchical confirmatory testing framework. Longer follow-up with repeated measurements would be needed to determine whether PVF responder status represents a sustained clinical benefit over time.

The combination of meloxicam and PCSO-524 did not demonstrate clear additional objective benefit over meloxicam alone within the 4-week study period. Although the combination group showed a numerically higher Week 4 responder proportion than placebo using the conservative >5%BW threshold, this comparison did not reach nominal statistical significance. The baseline-adjusted mean PVF analysis also failed to demonstrate additional objective limb-loading benefit of the combination treatment compared with meloxicam alone or PCSO-524 alone. Several explanations are possible. First, the short-term analgesic effect of meloxicam may have limited the ability to detect further objective improvement when PCSO-524 was added. Second, the 4-week treatment period may have been too short to detect the full objective effect of a marine-based fatty acid compound. Third, the study was not primarily powered to detect active-versus-active superiority between meloxicam alone and combination treatment. Therefore, the absence of clear additional PVF benefit should not be interpreted as evidence that combination treatment has no clinical value, but rather that objective superiority over meloxicam alone was not demonstrated under the conditions of this study.

Although combination treatment did not show a clear additional objective benefit based on PVF, it showed improvement in several owner-reported functional outcomes. For the owner-reported instruments used in this study, higher LOAD and CBPI pain severity and pain interference scores represented poorer clinical status. The CBPI quality-of-life item was reverse-coded as a separate impairment score, for which higher values indicated poorer quality of life. Consequently, lower scores indicated improvement across these measures, although changes in the CBPI quality-of-life item were interpreted cautiously because no within-group pairwise comparison remained significant after Bonferroni adjustment [9,33,34]. In the combination group, LOAD score decreased significantly from baseline to Week 4, indicating owner-perceived improvement in mobility and functional ability. CBPI pain interference score also decreased significantly from baseline to Week 4 and from Week 2 to Week 4, indicating that owners perceived less pain-related interference with daily activities. However, these owner-reported improvements were not accompanied by a nominally significant PVF responder advantage over placebo at either the conservative >5%BW threshold or the exploratory ≥3.5%BW threshold. This lack of concordance across outcome domains suggests that the observed owner-reported improvements should not be interpreted as evidence of objective treatment superiority. In addition, these findings should be interpreted in light of the questionnaire language and validation status: the Thai-language CBPI version had been used previously in a published clinical trial of canine osteoarthritis, whereas the Thai-language LOAD version was translated and clinically reviewed for the present study but has not been formally validated. These findings should be interpreted cautiously because owner-reported outcomes may be influenced by contextual and caregiver effects, even in blinded studies [11,35]. Nevertheless, they remain clinically relevant because owners assess their dogs in the home environment, where mobility, comfort, and daily function directly affect quality of life.

PCSO-524 showed significant within-group LOAD improvement at Week 2 and Week 4, suggesting a possible owner-perceived mobility benefit. PCSO-524 did not differ significantly from placebo using the conservative >5%BW PVF responder threshold. Using the exploratory ≥3.5%BW threshold, PCSO-524 showed a nominal Week 4 treatment-placebo responder signal; however, the overall treatment-group test for this exploratory threshold was not statistically significant, and no formal multiplicity adjustment was applied across responder thresholds, assessment time points, or treatment comparisons. Therefore, this PCSO-524 responder finding should be interpreted as exploratory and hypothesis-generating only. EAB-277 showed within-group improvement in CBPI pain interference from baseline to Week 4 but did not show a corresponding objective PVF responder advantage over placebo. Taken together, the differing patterns observed across treatment groups reinforce that statistically significant changes in individual secondary outcomes may not necessarily be corroborated by other measures of treatment response. Additional exploratory change-from-baseline analyses of LOAD and CBPI outcomes did not identify significant between-group differences among treatment groups at Week 2 or Week 4, further supporting cautious interpretation of these owner-reported findings. Because these owner-reported outcomes were secondary and because the Thai-language LOAD version has not undergone formal validation, the observed LOAD findings should be interpreted cautiously as owner-perceived functional changes rather than definitive evidence of treatment efficacy. These findings suggest that marine-based fatty acid compounds showed selected within-group improvements in owner-perceived domains of mobility or pain-related function, but short-term objective limb-loading improvement was not clearly demonstrated.

The veterinarian-assessed OAS did not change significantly in any treatment group. This score included lameness, articular mobility, and articular pain components, and may have been less sensitive to short-term treatment-associated changes than PVF responder status or owner-reported functional outcomes in this study. Four weeks of treatment may be insufficient to detect measurable changes in joint mobility, pain on manipulation, or examiner-assessed orthopedic impairment, particularly in dogs with chronic osteoarthritis. In addition, baseline OAS values were moderate on average, which may have limited the measurable range for improvement. The lack of OAS change, therefore, does not necessarily conflict with the secondary PVF responder signal or selected owner-reported findings. Rather, it suggests that veterinarian-assessed clinical examination scores may capture a dimension of treatment response that is less responsive to short-term change than objective limb-loading responder status or owner-reported functional assessment.

Short-term safety monitoring identified three withdrawals due to adverse events or intercurrent clinical conditions. Bloody diarrhea in one dog receiving combination treatment and increased serum creatinine concentration in one dog receiving meloxicam were considered clinically relevant events and possibly related to the study intervention, although treatment causality could not be definitively established. Pyometra in one placebo-treated dog was considered unrelated to the study intervention. These events did not indicate a consistent group-level safety signal, but the 4-week treatment duration and limited sample size restrict conclusions regarding uncommon, delayed, or chronic adverse effects. Therefore, the safety findings should be interpreted as short-term safety and tolerability data rather than evidence of long-term or chronic-treatment safety.

Clinically, these findings suggest that short-term treatment response in dogs with hip osteoarthritis should be assessed across multiple dimensions [8,36]. Meloxicam showed a nominal supportive individual-dog PVF responder signal at Week 4 and may be clinically relevant when the treatment goal is short-term improvement in objective limb loading. However, this responder finding should not be interpreted as confirmatory evidence of overall treatment superiority. Combination treatment did not demonstrate additional objective benefit over meloxicam alone but showed selected within-group improvements in owner-reported functional outcomes, suggesting potential relevance when owner-perceived mobility and pain-related daily activity are important treatment targets. Marine-based fatty acid compounds showed selected owner-reported improvements, and PCSO-524 showed an exploratory PVF responder signal only at the lower ≥3.5%BW threshold; therefore, expectations regarding short-term objective gait improvement should remain cautious. Overall, treatment decisions should integrate objective gait assessment, owner-reported functional outcomes, clinical examination findings, and safety monitoring rather than relying on a single outcome measure.

This study has several strengths. It used a randomized, double-blind, placebo-controlled design in a clinically relevant population of client-owned dogs with naturally occurring hip osteoarthritis. The study evaluated multiple clinically relevant treatment approaches using both objective force-plate analysis and owner-reported outcome measures. This design allowed comparison of objective limb-loading response, owner-perceived functional change, and short-term laboratory safety within the same clinical trial. The inclusion of a secondary responder analysis enhanced the clinical interpretability of the PVF data by identifying individual dogs that achieved a conservative analysis-stage threshold of PVF improvement [12].

Several limitations should be considered. The treatment period was limited to 4 weeks, which may have been sufficient to detect short-term NSAID-associated effects but may not have fully captured the time course of response to marine-based fatty acid compounds. Longer-term studies are needed to determine whether the selected owner-reported improvements observed in this study persist, increase, or translate into objective limb-loading improvement over time. The study was powered based on force-plate measurements and was not primarily powered to detect active-versus-active superiority, particularly between meloxicam alone and combination treatment, or to detect differences in secondary responder and owner-reported outcomes. Radiographic assessment was used only to confirm eligibility and was not used as an outcome measure, severity-stratification variable, or covariate in the efficacy analyses. Formal radiographic grading of hip dysplasia or osteoarthritis severity was not performed; therefore, the study could not evaluate whether radiographic severity modified treatment response. Although an exploratory severity-adjusted analysis did not identify evidence of a treatment group-by-severity interaction, the study was not powered primarily to evaluate severity-based subgroup effects.

An additional limitation was a deviation from the intended implementation of the stratified block randomization procedure. Although allocation positions reserved for dogs who did not attend their scheduled pretreatment visits should have been made available for subsequent eligible dogs within the same severity stratum, these positions were not reassigned because of an administrative coordination oversight. Consequently, some randomization blocks remained incomplete, resulting in unequal treatment-group sizes within the severity strata.

The deviation did not appear to involve disclosure of treatment allocation information to the investigators responsible for recruitment and outcome assessment. The randomization schedules were generated in advance and maintained exclusively by an independent investigator, and the clinical investigators responsible for recruitment and outcome assessment had no access to the schedules or treatment codes. The unfilled positions arose before any study medication was dispensed, and full reconciliation confirmed that all 203 documented allocations corresponded to the treatment-group distributions reported in Table 2. Baseline characteristics, including severity classification, were comparable among groups. Nevertheless, because implementation departed from the intended procedure, a residual risk of selection bias cannot be entirely excluded.

The pain-related rescue-withdrawal criterion also warrants consideration. No pain-related rescue withdrawal was recorded under this criterion, and the rescue protocol was not applied to any dog. However, the predefined threshold, a ≥3-point increase in the CBPI Pain Interference Score, was designed to detect deterioration from each dog’s baseline rather than to define an absolute level of clinically unacceptable pain. Dogs with considerable pain at enrolment may therefore have experienced clinically relevant discomfort without reaching this threshold. This concern applies mainly to dogs in the severe bilateral hip pain stratum allocated to placebo or marine-based fatty acid treatment without concurrent NSAID therapy. To mitigate this risk, the study combined scheduled clinical assessments with owner-initiated reporting via telephone and project-specific messaging channels, the option of voluntary withdrawal, and ongoing access to veterinary care. To capture such cases more fully, future studies may benefit from combining a deterioration-based criterion with an absolute pain threshold, so that rescue intervention is triggered either by marked worsening from baseline or by pain exceeding a predefined level, regardless of the starting point.

For dogs with bilateral hip osteoarthritis, the painful hindlimb with the lower baseline PVF was selected as the index limb. Although this approach was intended to identify the more functionally affected limb and to maintain a consistent limb-specific outcome, it may have introduced regression to the mean. A limb selected because of a relatively low baseline PVF may show some increase on repeat measurement independent of treatment, particularly in responder analyses based on change from baseline. The primary baseline-adjusted linear mixed model included baseline PVF as a covariate, which helped account for baseline limb-loading differences in the mean PVF analysis; however, the secondary responder analyses remained potentially susceptible to regression-to-the-mean effects and were therefore interpreted cautiously. The >5%BW responder threshold was selected during the statistical analysis stage based on published recommendations and should be regarded as an analysis-stage responder definition rather than a protocol-specified criterion established before recruitment. This threshold was not intended to represent a formally validated minimal clinically important difference in this study population. Although Bonferroni adjustment was applied for pairwise comparisons within individual outcomes, no formal multiplicity adjustment was applied across all outcome measures. In addition, no hierarchical confirmatory testing framework or formal multiplicity adjustment was applied across responder thresholds, assessment time points, or treatment comparisons. Because multiple clinical and laboratory outcomes and exploratory analyses were evaluated, including the exploratory ≥3.5%BW responder threshold, isolated significant findings may have arisen by chance and should therefore be interpreted cautiously.

The owner-reported outcomes also have instrument-related limitations. Although the Thai-language CBPI version had prior published use in canine osteoarthritis, the Thai-language LOAD version was translated and clinically reviewed for the present study but has not been formally validated. Owners were instructed to have the same owner or primary caregiver complete the questionnaires whenever possible, but this should be interpreted as a study instruction rather than a formally verified criterion for every questionnaire. In addition, baseline CBPI pain severity scores were relatively low, which may have produced a floor effect and limited the ability to detect further measurable improvement in owner-reported pain severity. Owner-reported outcomes may be influenced by caregiver expectations or contextual effects, even in a blinded trial. In addition, this was a single-center study conducted in client-owned medium- to large-sized dogs with naturally occurring hip osteoarthritis, predominantly represented by retriever-type breeds and Siberian Huskies; therefore, generalizability to dogs with different breed, conformational, or osteoarthritis phenotypes should be considered cautiously. The trial was not prospectively registered in a public clinical trial registry, which should be considered a transparency limitation. Finally, efficacy analyses used a modified intention-to-treat approach with available-case analysis, and missing post-baseline data were not imputed. These limitations should be considered when interpreting both the objective and owner-reported findings.

## 5. Conclusions

In this population of client-owned dogs with naturally occurring hip osteoarthritis, the primary baseline-adjusted mean PVF analysis failed to demonstrate superiority of any active treatment over placebo over the 4-week treatment period; therefore, secondary responder and score-based findings should be interpreted in this context. The analysis also failed to demonstrate additional objective limb-loading benefit of the meloxicam and PCSO-524 combination compared with either component alone. Meloxicam showed a nominal supportive individual-dog PVF responder signal at Week 4 using a conservative analysis-stage >5%BW threshold; however, this secondary responder finding should be interpreted as supportive rather than confirmatory. Secondary score-based outcomes showed selected within-group improvements in owner-reported LOAD and CBPI domains, but these findings were not consistently supported by between-group differences versus placebo and should therefore be interpreted as supportive rather than confirmatory. Short-term laboratory safety findings did not identify consistent group-level hematological or serum biochemical abnormalities over 4 weeks, but these findings should not be interpreted as evidence of safety for chronic treatment. Overall, these findings reinforce that treatment response in canine hip osteoarthritis should not be judged using a single outcome measure. Objective gait analysis, responder-based interpretation, owner-reported functional assessment, clinical examination, and safety monitoring each provide different but complementary information for clinical management.

## Figures and Tables

**Table 1:** Orthopedic assessment score (OAS) used for clinical evaluation of dogs with hip osteoarthritis.

Clinical Parameter	Score	Scoring Criterion
**Lameness** |*Scored once per dog*		
**Lameness**	**0**	Stands, walks, and trots normally
	**1**	Stands normally, with slight algetic gait when trotting
	**2**	Stands normally, with slight algetic gait when walking
	**3**	Stands normally, with evident algetic gait when walking
	**4**	Stands abnormally, with evident algetic gait when trotting
**Articular mobility** |*Scored separately for each hip*		
**Articular mobility**	**0**	No limitation of movement or crepitus
	**1**	10% to 20% decrease in ROM, without crepitus
	**2**	10% to 20% decrease in ROM, with crepitus
	**3**	20% to 50% decrease in ROM
	**4**	More than 50% decrease in ROM
**Articular pain** |*Scored separately for each hip*		
**Articular pain**	**0**	No sign of pain
	**1**	Mild pain; dog turns head in recognition
	**2**	Moderate pain; dog pulls limb away or wants to move away
	**3**	Severe pain; dog vocalizes and becomes aggressive

Note: OAS = orthopedic assessment score; ROM = range of motion. Lameness was scored once per dog. Articular mobility and articular pain were scored for each hip. The total OAS was calculated as the sum of the lameness score, left and right hip articular mobility scores, and left and right hip articular pain scores, giving a total possible score of 0 to 18. Higher scores indicate greater orthopedic impairment. Adapted from Moreau et al., 2003 [6].

**Table 2:** Baseline demographic and clinical characteristics of dogs with hip osteoarthritis by treatment group.

Characteristic	Combination (*n* = 41)	Meloxicam (*n* = 41)	Placebo (*n* = 40)	PCSO-524 (*n* = 41)	EAB-277 (*n* = 40)	*p* Value
Disease severity, n (%)						0.889
Mild-to-moderate	21 (51.2)	21 (51.2)	23 (57.5)	21 (51.2)	21 (52.5)	
Severe	20 (48.8)	20 (48.8)	17 (42.5)	20 (48.8)	19 (47.5)	
General demographic variables						
Age, months, mean ± SD	50.0 ± 20.2	60.8 ± 30.7	64.8 ± 26.9	61.8 ± 32.8	65.6 ± 33.8	0.121
Body weight, kg, mean ± SD	35.9 ± 6.3	35.1 ± 9.7	35.1 ± 6.4	34.0 ± 7.5	32.3 ± 5.3	0.200
Body condition score, *n* (%)						0.088
3/5	18 (43.9)	17 (41.5)	11 (27.5)	13 (31.7)	25 (62.5)	
4/5	16 (39.0)	17 (41.5)	22 (55.0)	18 (43.9)	12 (30.0)	
5/5	7 (17.1)	7 (17.1)	7 (17.5)	10 (24.4)	3 (7.5)	
Sex, *n* (%)						0.500
Female	15 (36.6)	17 (41.5)	20 (50.0)	14 (34.1)	13 (32.5)	
Male	26 (63.4)	24 (58.5)	20 (50.0)	27 (65.9)	27 (67.5)	
Breed, *n* (%)						0.628
Alaskan Malamute	3 (7.3)	1 (2.4)	1 (2.5)	2 (4.9)	0 (0.0)	
American Pit Bull Terrier	2 (4.9)	1 (2.4)	1 (2.5)	0 (0.0)	2 (5.0)	
Crossbreed	1 (2.4)	2 (4.9)	2 (5.0)	2 (4.9)	1 (2.5)	
German Shepherd Dog	1 (2.4)	0 (0.0)	0 (0.0)	0 (0.0)	2 (5.0)	
Golden Retriever	19 (46.3)	23 (56.1)	19 (47.5)	15 (36.6)	20 (50.0)	
Labrador Retriever	10 (24.4)	8 (19.5)	9 (22.5)	11 (26.8)	4 (10.0)	
Rottweiler	1 (2.4)	1 (2.4)	2 (5.0)	1 (2.4)	0 (0.0)	
Siberian Husky	4 (9.8)	5 (12.2)	5 (12.5)	8 (19.5)	10 (25.0)	
Thai Bangkaew Dog	0 (0.0)	0 (0.0)	1 (2.5)	2 (4.9)	0 (0.0)	
Thai Ridgeback Dog	0 (0.0)	0 (0.0)	0 (0.0)	0 (0.0)	1 (2.5)	
Baseline clinical outcome measures						
PVF, %BW, mean ± SD	65.54 ± 7.68	64.64 ± 9.31	65.84 ± 8.57	65.65 ± 8.54	66.38 ± 6.69	0.917
Orthopedic assessment score (OAS), mean ± SD	7.22 ± 3.57	6.63 ± 2.90	6.45 ± 3.57	6.85 ± 3.23	6.77 ± 3.04	0.816
LOAD score, median [IQR]	15.00 [11.75, 19.25]	14.00 [8.00, 17.50]	14.00 [8.00, 20.00]	15.00 [11.00, 21.00]	11.00 [9.00, 16.50]	0.171
CBPI pain severity score, median [IQR]	1.25 [0.00, 2.81]	0.88 [0.00, 2.13]	0.25 [0.00, 2.19]	0.50 [0.00, 2.25]	0.25 [0.00, 1.25]	0.443
CBPI pain interference score, median [IQR]	2.17 [0.63, 4.21]	1.42 [0.00, 2.83]	1.00 [0.04, 2.96]	1.00 [0.00, 3.08]	1.00 [0.17, 2.50]	0.275
CBPI quality-of-life impairment score (reverse-coded), median [IQR]	3.00 [2.00, 3.25]	3.00 [2.00, 3.00]	3.00 [2.00, 3.00]	3.00 [2.00, 3.00]	2.00 [2.00, 3.00]	0.525

Note: Values are presented as mean ± SD, *n* (%), or median [interquartile range], as appropriate. PVF = peak vertical force of the index hindlimb; %BW = percentage of body weight; OAS = orthopedic assessment score; LOAD = Liverpool Osteoarthritis in Dogs questionnaire; CBPI = Canine Brief Pain Inventory; IQR = interquartile range; SD = standard deviation. The CBPI quality-of-life item was reverse-coded as an impairment score, ranging from 1 (excellent) to 5 (poor); higher scores indicate poorer quality of life.

**Table 3:** Baseline-adjusted estimated marginal means of PVF and within-group comparisons between Week 2 and Week 4.

Treatment Group	Week 2 Adjusted Mean (%BW)	Week 4 Adjusted Mean (%BW)	Mean Difference	95% CI	Adjusted *p*-Value
Combination	67.98	68.23	0.25	−0.79 to 1.29	0.639
Meloxicam	66.74	68.31	1.57	0.53 to 2.62	**0.003**
Placebo	66.53	66.30	−0.23	−1.29 to 0.83	0.670
PCSO-524	66.75	67.49	0.74	−0.29 to 1.76	0.157
EAB-277	67.22	67.01	−0.21	−1.25 to 0.83	0.689

Note: Values are baseline-adjusted estimated marginal means from the baseline-adjusted linear mixed model. Mean differences represent Week 4 minus Week 2. *p*-values were adjusted using the Bonferroni method. PVF = peak vertical force of the index hindlimb; %BW = percentage of body weight; CI = confidence interval.

**Table 4:** Responder analysis of PVF improvement at Weeks 2 and 4 using an absolute >5%BW threshold.

Time Point	Treatment Group	Responders/Total	Responder Rate (%)	RR vs. Placebo	95% CI	*p*-Value
**Week 2**	***Placebo***	***5/39***	***12.8***	***Reference***		
	Combination	11/41	26.8	2.093	0.800 to 5.476	0.132
	Meloxicam	4/41	9.8	0.761	0.220 to 2.628	0.666
	PCSO-524	2/41	4.9	0.380	0.078 to 1.848	0.231
	EAB-277	6/40	15.0	1.170	0.389 to 3.521	0.780
**Week 4**	***Placebo***	***3/39***	***7.7***	***Reference***		
	Combination	10/39	25.6	3.333	0.993 to 11.195	*0.051*
	Meloxicam	12/39	30.8	4.000	1.223 to 13.079	**0.022**
	PCSO-524	6/41	14.6	1.902	0.511 to 7.084	0.338
	EAB-277	8/40	20.0	2.600	0.744 to 9.088	0.135

Note: PVF responder status was defined as an absolute increase in PVF of >5%BW from baseline at each time point; dogs with an increase of ≤5%BW were classified as non-responders. This conservative PVF-based threshold was selected during the statistical analysis stage and was informed by published recommendations for interpreting ground reaction force changes and measurement variability in dogs [12]. The threshold was not derived from a study-specific test-retest measurement-error estimate and should not be interpreted as a formally validated minimal clinically important difference. Relative risks were estimated using modified Poisson regression with a log link and robust covariance estimation, with placebo as the reference group. CI = confidence interval; PVF = peak vertical force of the index hindlimb; RR = relative risk; %BW = percentage of body weight.

**Table 5:** Changes in LOAD score over the 4-week treatment period.

Treatment Group	Baseline	Week 2	Week 4	Friedman *p*-Value	Significant Pairwise Comparison
Combination	15.00 [11.75, 19.25]	15.00 [12.00, 19.00]	13.00 [7.00, 19.50]	**0.016**	**Baseline to Week 4, *p* = 0.013**
Meloxicam	14.00 [8.00, 17.50]	12.00 [9.00, 17.00]	12.00 [5.00, 18.50]	0.097	None
Placebo	14.00 [8.00, 20.00]	15.00 [9.00, 21.00]	14.00 [7.00, 20.75]	0.051	None
PCSO-524	15.00 [11.00, 21.00]	12.00 [7.75, 16.00]	11.00 [8.00, 16.50]	**0.005**	**Baseline to Week 2, *p* = 0.018; Baseline to Week 4, *p* = 0.023**
EAB-277	11.00 [9.00, 16.50]	11.00 [8.00, 16.00]	9.00 [8.00, 16.00]	**0.038**	None after Bonferroni correction

Note: Data are presented as median [interquartile range]. Higher LOAD scores indicate poorer mobility and functional ability; decreases from baseline indicate improvement. Within-group changes were analyzed using Friedman tests, with Bonferroni-adjusted pairwise comparisons. LOAD = Liverpool Osteoarthritis in Dogs questionnaire.

**Table 6:** Changes in CBPI pain-domain scores and the reverse-coded global quality-of-life item over the 4-week treatment period.

CBPI Measure	Treatment Group	Baseline	Week 2	Week 4	Friedman *p*-Value	Significant Pairwise Comparison
**Pain severity**	Combination	1.25 [0.00, 2.81]	1.00 [0.00, 2.75]	0.75 [0.00, 2.13]	0.321	None
	Meloxicam	0.88 [0.00, 2.13]	1.00 [0.25, 2.38]	1.00 [0.00, 2.38]	0.191	None
	Placebo	0.25 [0.00, 2.19]	1.00 [0.00, 1.75]	0.75 [0.00, 1.75]	0.627	None
	PCSO-524	0.50 [0.00, 2.25]	0.25 [0.00, 1.00]	0.50 [0.00, 1.50]	**0.014**	None after Bonferroni correction
	EAB-277	0.25 [0.00, 1.25]	0.00 [0.00, 1.00]	0.25 [0.00, 1.75]	**0.039**	None after Bonferroni correction
**Pain interference**	Combination	2.17 [0.63, 4.21]	1.75 [0.17, 3.29]	0.67 [0.00, 2.33]	**0.004**	**Baseline to Week 4, *p* = 0.009; Week 2 to Week 4, *p* = 0.029**
	Meloxicam	1.42 [0.00, 2.83]	1.33 [0.17, 2.92]	1.00 [0.00, 2.08]	**0.032**	None after Bonferroni correction
	Placebo	1.00 [0.04, 2.96]	1.17 [0.00, 2.33]	0.67 [0.00, 2.17]	0.149	None
	PCSO-524	1.00 [0.00, 3.08]	0.33 [0.00, 1.29]	0.33 [0.00, 1.33]	0.211	None
	EAB-277	1.00 [0.17, 2.50]	0.50 [0.00, 1.83]	0.17 [0.00, 1.75]	**0.008**	**Baseline to Week 4, *p* = 0.024**
**Quality-of-life impairment (reverse-coded)**	Combination	3.00 [2.00, 3.25]	3.00 [2.00, 4.00]	2.00 [1.50, 3.00]	**0.009**	None after Bonferroni correction
	Meloxicam	3.00 [2.00, 3.00]	2.00 [2.00, 3.00]	2.00 [2.00, 3.00]	0.662	None
	Placebo	3.00 [2.00, 3.00]	3.00 [2.00, 3.00]	3.00 [2.00, 3.00]	0.685	None
	PCSO-524	3.00 [2.00, 3.00]	3.00 [2.00, 3.00]	3.00 [2.00, 3.00]	0.840	None
	EAB-277	2.00 [2.00, 3.00]	2.00 [2.00, 3.00]	2.00 [2.00, 3.00]	0.984	None

Note: Values are presented as median [IQR]. Higher CBPI pain severity and pain interference scores indicate greater pain severity and pain-related functional impairment. The CBPI quality-of-life item was reverse-coded as a QOL impairment score ranging from 1 (excellent) to 5 (poor), with higher values indicating poorer QOL. Decreases in the respective scores indicate improvement. Descriptive statistics were calculated from available observations at each visit, whereas Friedman tests included only dogs with complete data for the corresponding CBPI measure at all three assessment times. Within-group changes were evaluated using Friedman tests, with Bonferroni-adjusted pairwise comparisons. CBPI = Canine Brief Pain Inventory; QOL = quality of life; IQR = interquartile range.

## Data Availability

The data presented in this study are available within the article. Raw data supporting this study are available from the corresponding author upon reasonable request.

## Supplementary Materials

The following supporting information can be downloaded at: https://www.mdpi.com/article/10.3390/ani16213435/s1. Supplementary Table S1: Composition, concentration, and batch information of the active study medications. Supplementary Table S2: Baseline-adjusted Week 4 PVF comparisons addressing the primary study objectives. Supplementary Table S3: Paired individual changes in index-hindlimb peak vertical force from baseline to Weeks 2 and 4. Supplementary Table S4: Exploratory responder analysis of PVF improvement at Weeks 2 and 4 using an absolute ≥3.5%BW threshold.

## Abbreviations

The following abbreviations are used in this manuscript:

ALB	albumin
ALP	alkaline phosphatase
ALT	alanine aminotransferase
ANCOVA	analysis of covariance
BCS	body condition score
BUN	blood urea nitrogen
BW	body weight
CBC	complete blood count
CBPI	Canine Brief Pain Inventory
CI	confidence interval
COX	cyclooxygenase
EAB-277	stabilized lipid extract containing green-lipped mussel and krill lipid
GRF	ground reaction force
IACUC	Institutional Animal Care and Use Committee
QR	interquartile range
LOAD	Liverpool Osteoarthritis in Dogs
NSAID	non-steroidal anti-inflammatory drug
OA	osteoarthritis
OAS	orthopedic assessment score
PCSO-524	stabilized lipid extract derived from New Zealand green-lipped mussel
PVF	peak vertical force
ROM	range of motion
RR	relative risk
SD	standard deviation
SPSS	Statistical Package for the Social Sciences
WBC	white blood cell
%BW	percentage of body weight
