# Supplementary material for: Short-Term Effects of Meloxicam and Marine-Based Fatty Acid Compounds on Objective Limb Loading and Owner-Reported Outcomes in Dogs with Hip Osteoarthritis: A Randomized, Double-Blind, Placebo-Controlled Trial
Source: Animals (Basel). 2026 Oct 30;16(21):3435. doi: 10.3390/ani16213435 (PMC13762341; doi:10.3390/ani16213435)
Supplement: Supplementary file 1 [file animals-16-03435-s001.zip › animals-4589859-supplementary/Supplementary Table S1. Composition and batch information of the medications used in the study^LL.pdf]

Supplementary Table S1. Composition, concentration, and batch information of the active study medications

Medication	Composition / concentration	Batch number
**Meloxicam**	Meloxicam 0.15%	NA
**PCSO-524**	PCSO-524 50 mg, olive oil 100 mg, and d-alpha-tocopherol 0.225 mg	AL46424
**EAB-277**	New Zealand green-lipped mussel oil 30 mg, high-phospholipid krill oil 20 mg, and olive oil 100 mg	NHB0822

Note: Ingredient quantities for PCSO-524 and EAB-277 are reported per capsule; the listed ingredients do not represent the complete formulation. NA = batch number not available.
