# Supplementary material for: Short-Term Effects of Meloxicam and Marine-Based Fatty Acid Compounds on Objective Limb Loading and Owner-Reported Outcomes in Dogs with Hip Osteoarthritis: A Randomized, Double-Blind, Placebo-Controlled Trial
Source: Animals (Basel). 2026 Oct 30;16(21):3435. doi: 10.3390/ani16213435 (PMC13762341; doi:10.3390/ani16213435)
Supplement: Supplementary file 1 [file animals-16-03435-s001.zip › animals-4589859-supplementary/Supplementary Table S2 Baseline-adjusted Week 4 PVF comparisons addressing the primary study objectives.pdf]

Supplementary Table S2. Baseline-adjusted Week 4 PVF comparisons addressing the primary study objectives.

Week 4 comparison	Adjusted mean difference, %BW	95% CI	Bonferroni-adjusted p-value
**Combination vs placebo**	1.94	−0.51 to 4.38	0.259
**Meloxicam vs placebo**	2.01	−0.43 to 4.46	0.206
**PCSO-524 vs placebo**	1.19	−1.23 to 3.62	1.000
**EAB-277 vs placebo**	0.72	−1.73 to 3.16	1.000
**Combination vs meloxicam**	−0.08	−2.52 to 2.36	1.000
**Combination vs PCSO-524**	0.74	−1.68 to 3.16	1.000

Note: Values are baseline-adjusted mean differences from the primary linear mixed model for PVF at Week 4. Positive values indicate higher PVF in the first-listed treatment group. P-values were adjusted using the Bonferroni method. CI = confidence interval; PVF = peak vertical force; %BW = percentage of body weight.
