# Supplementary material for: Short-Term Effects of Meloxicam and Marine-Based Fatty Acid Compounds on Objective Limb Loading and Owner-Reported Outcomes in Dogs with Hip Osteoarthritis: A Randomized, Double-Blind, Placebo-Controlled Trial
Source: Animals (Basel). 2026 Oct 30;16(21):3435. doi: 10.3390/ani16213435 (PMC13762341; doi:10.3390/ani16213435)
Supplement: Supplementary file 1 [file animals-16-03435-s001.zip › animals-4589859-supplementary/Supplementary Table S3. Paired individual changes in index-hindlimb peak vertical force from baseline to Weeks 2 and 4.pdf]

Supplementary Table S3. Paired individual changes in index-hindlimb peak vertical force from baseline to Weeks 2 and 4

Treatment group	Week 2 N	Week 2 mean ± SD	Week 2 median [IQR]	Week 2 min to max	Week 4 N	Week 4 mean ± SD	Week 4 median [IQR]	Week 4 min to max
Combination	41	2.44 ± 4.18	2.37 [−0.83, 5.61]	−8.15 to 10.38	39	2.59 ± 4.71	1.11 [−0.69, 5.31]	−5.21 to 15.60
Meloxicam	41	1.28 ± 3.84	1.63 [−0.65, 3.21]	−8.82 to 10.22	39	3.07 ± 4.32	2.18 [0.45, 5.57]	−7.75 to 10.92
Placebo	39	0.95 ± 3.87	0.83 [−1.57, 3.73]	−7.06 to 9.33	39	0.69 ± 3.19	0.71 [−1.41, 2.98]	−5.29 to 8.17
PCSO-524	41	1.20 ± 2.73	1.27 [−0.73, 3.62]	−5.66 to 5.55	41	1.94 ± 3.93	2.26 [−0.86, 4.52]	−7.10 to 14.83
EAB-277	40	1.60 ± 3.33	1.27 [0.20, 3.27]	−7.27 to 8.94	40	1.39 ± 4.57	1.32 [−1.55, 4.31]	−15.02 to 12.50

Note: Values represent paired individual changes in PVF, calculated as post-treatment PVF minus baseline PVF for each dog. Positive values indicate increased PVF from baseline. Values are reported as mean ± SD, median [interquartile range], and minimum to maximum. PVF = peak vertical force; %BW = percentage of body weight.
