# Supplementary material for: Short-Term Effects of Meloxicam and Marine-Based Fatty Acid Compounds on Objective Limb Loading and Owner-Reported Outcomes in Dogs with Hip Osteoarthritis: A Randomized, Double-Blind, Placebo-Controlled Trial
Source: Animals (Basel). 2026 Oct 30;16(21):3435. doi: 10.3390/ani16213435 (PMC13762341; doi:10.3390/ani16213435)
Supplement: Supplementary file 1 [file animals-16-03435-s001.zip › animals-4589859-supplementary/Supplementary Table S4 Exploratory responder analysis of PVF improvement at weeks 2 and 4 using an absolute ≥3.5 ^1BW threshold^.pdf]

Supplementary Table S4. Exploratory responder analysis of PVF improvement at Weeks 2 and 4 using an absolute ≥3.5 %BW threshold.

Time point	Treatment group	Responders / total	Responder rate (%)	RR vs placebo	95% CI	p-value
**Week 2**	Placebo	11/39	28.2	**Reference**		
	Combination	16/41	39.0	1.384	0.737 to 2.598	0.313
	Meloxicam	8/41	19.5	.692	0.311 to 1.537	0.366
	PCSO-524	11/41	26.8	.951	0.467 to 1.938	0.890
	EAB-277	8/40	20.0	.709	0.320 to 1.573	0.398
**Week 4**	Placebo	7/39	17.9	**Reference**		
	Combination	13/39	33.3	1.857	0.831 to 4.152	0.132
	Meloxicam	17/39	43.6	2.429	1.136 to 5.193	0.022
	PCSO-524	16/41	39.0	2.174	1.004 to 4.707	0.049
	EAB-277	10/40	25.0	1.393	0.590 to 3.289	0.450

Note: PVF responder status was defined as an absolute increase in PVF of ≥3.5 %BW from baseline at each post-treatment assessment. This lower threshold was evaluated as an exploratory sensitivity analysis, informed by published consensus recommendations regarding clinically important changes in canine ground reaction forces. Relative risks were estimated using modified Poisson regression with a log link and robust covariance estimation, with placebo as the reference group. P-values were not adjusted for study-wide multiplicity and should therefore be interpreted as exploratory. **Overall treatment-group tests from the same modified Poisson regression model showed robust Wald p-values of 0.255 at Week 2 and 0.131 at Week 4.** CI = confidence interval; PVF = peak vertical force of the index hindlimb; RR = relative risk; %BW = percentage of body weight.
